# Transferability and characterization of microsatellite markers in two Neotropical *Ficus* species

**DOI:** 10.1590/S1415-47572009005000056

**Published:** 2009-09-01

**Authors:** Alison Gonçalves Nazareno, Rodrigo Augusto Santinelo Pereira, Juliana Massimino Feres, Moacyr Antônio Mestriner, Ana Lilia Alzate-Marin

**Affiliations:** Programa de Pós-graduação em Biologia Comparada, Faculdade de Filosofia, Ciências e Letras de Ribeirão Preto, Universidade de São Paulo, Ribeirão Preto, SPBrazil; 2 Departamento de Biologia, Faculdade de Filosofia, Ciências e Letras de Ribeirão Preto, Universidade de São Paulo, Ribeirão Preto, SPBrazil; 3 Laboratório de Genética Vegetal, Departamento de Genética, Faculdade de Medicina de Ribeirão Preto, Universidade de São Paulo, Ribeirão Preto, SPBrazil

**Keywords:** ecological genetics, *Ficus citrifolia*, *Ficus eximia*, Moraceae, SSRs

## Abstract

Microsatellite markers were transferred and characterized for two Neotropical fig tree species, *Ficus citrifolia* and *Ficus eximia*. Our study demonstrated that microsatellite markers developed from different subgenera of *Ficus* can be transferred to related species. In the present case, 12 of the 15 primer pairs tested (80%) were successfully transferred to both of the above species. Eleven loci were polymorphic when tested across 60 *F. citrifolia* and 60 *F. eximia* individuals. For *F. citrifolia*, there were 4 to 15 alleles per locus, whereas expected heterozygosities ranged from 0.31 to 0.91. In the case of *F. eximia*, this was 2 to 12 alleles per locus and expected heterozygosities from 0.42 to 0.87.

Effective strategies for the conservation of genetic resources in tropical forests are of great importance, mainly due to the negative impacts arising from the reduction in biological diversity. This is especially true with regard to ecologically important species such as fig trees (*Ficus* species, family Moraceae), which are considered to be keystone- resources in tropical forests, through supplying frugivores with fruit during periods of food-scarcity (Shanahan *et al.*, 2001). Furthermore, plants of the genus *Ficus* are considered as a classic example of plant-insect mutualism (Weiblen, 2002). With few exceptions, each of the 750 *Ficus* species maintains an obligatory symbiotic interaction with a specific pollinating wasp species (Hymenoptera: Agaonidae). However, little is known about the genetic diversity and population structure of *Ficus* species (Dick *et al.*, 2008)*.* Microsatellite markers (simple sequence repeats - SSR) are informative tools used to assess the genetic structure of populations as well as basic quantitative genetic parameters. Although microsatellite markers constitute informative systems for ecological genetics, they have only been isolated for seven of the 750 species of *Ficus* (Khadari *et al.*, 2001; Giraldo *et al.*, 2005; Zavodna *et al.*, 2005; Vignes *et al.*, 2006; Ahmed *et al.*, 2007; Bandelj *et al.*, 2007; Crozier *et al.*, 2007). Nevertheless, the high transferability of these markers has allowed for cross amplification in 47 *Ficus* species (Khadari *et al.*, 2001; Giraldo *et al.*, 2005; Vignes *et al.*, 2006). Moreover, indications of high transferability within a particular genus has also come to light from other areas of research (Poncet *et al.*, 2004; Moon *et al.*, 2008). Given the time consuming and relatively costly process of isolating microsatellites and the low frequency of SSRs in plants (Powell *et al.*, 1996), it is a decided advantage to be able to utilize primer sequences identified in one species in other closely related ones. Here, we examine the transferability and the characterization of microsatellite markers previously developed from different subgenera of *Ficus* for two species occurring in Brazil, *Ficus citrifolia* P. Miller and *Ficus eximia* Schott.

The subgenus *Urostigma* section *Americana*, to which *F. citrifolia* and *F. eximia* belong, includes monoecious plants that may occur as trees of hemi-epiphyte growth form (Berg, 1989). *Ficus citrifolia* normally grows as a hemiepiphyte on other trees or buildings and frequently develops within disturbed areas. *Ficus eximia* usually germinates on fallen trunks and grows as a free-standing tree in humid patches in the forest. During January and February, 2008, we sampled 120 individuals from two natural populations (30 individuals per area per species), 350 km apart, located at the Parque Estadual Morro do Diabo (22° 27' - 22° 40' S, 52° 10' - 52° 22' W) and at the Estação Ecológica de Caetetus (22° 41' - 22° 46' S, 49° 10' - 49° 16' W), both in southeastern Brazil.

DNA for microsatellite analysis was extracted from frozen leaves by using the cetyltrimethyl ammonium bromide (CTAB) extraction method (Doyle and Doyle, 1990). Fifteen microsatellite loci, previously developed for *Ficus* (*Pharmacosycea*) *insipida* (Vignes *et al.*, 2006), *Ficus* (*Sycomorus*) *racemosa* and *Ficus* (*Urostigma*) *rubiginosa* (Crozier *et al.*, 2007), were tested for cross amplification in specimens of *F. citrifolia* and *F. eximia*. Using polymerase chain reaction (PCR), a screening of each primer pair through ten annealing temperatures (between 46-55 °C) was accomplished with 10 individuals of *F. citrifolia* and *F. eximia*. Microsatellite loci were amplified in a final volume of 10 μL containing 0.3 μM of each primer, 1 U *Taq* DNA polymerase, 0.25 mM of each dNTP, 1 x MgCl_2_-free reaction buffer [75 mM Tris-HCl pH 9.0, 50 mM KCl and 20 mM (NH_4_)_2_SO_4_], 1.5 mM MgCl_2_ and 2.5 ng of template DNA. The amplification was performed using a MasterCycler Eppendorf under the following conditions: 5 min of denaturation at 96 °C and 30 cycles of 30 s of initial denaturation at 94 °C, 1 min of annealing at *T*_a_ (Table 1) and 1 min of extension at 72 °C, to finish with 7 min of elongation at 72 °C. Amplified fragments were separated on 10% denaturing polyacrylamide gels in 8 M urea and 1 x TBE buffer, to then be stained with silver nitrate (Sanguinetti *et al.*, 1994). The quantification of allele size was scored against a 10 bp DNA ladder standard (Invitrogen).

Genetic diversity parameters and probabilities of paternity exclusion were estimated using CERVUS version 3.0 (Kalinowski *et al.*, 2007). The FSTAT software package version 1.2 (Goudet, 2001) was used to test all loci for linkage disequilibrium, with application of Bonferroni correction for multiple comparisons.

Our study demonstrated that microsatellite markers developed from different subgenera of *Ficus* can be transferred to related species. We successfully transferred 12 of the 15 primer pairs tested (80%) to both of the species, *F. citrifolia* and *F. eximia* (Table 1). Similar allele numbers and length of amplification products were apparent in most of the successful loci, when compared with the species from which they were developed (Table 1). Of the 12 loci transferred, 11 were polymorphic for both *F. citrifolia* and *F. eximia*. Loci FinsT7 and Frub154 were monomorphic in only one species. Eighty-seven *F. citrifolia* and 77 *F. eximia* allelic variants were identified (Table 1). Furthermore, the average for heterozygosity and the mean number of alleles per loci were, respectively, 0.67 and 7.3 in *F. citrifolia* and 0.69 and 6.4 in *F. eximia*. Heterozygosity values of *F. citrifolia* and *F. eximia* in this study were within those for *Ficus* species reported in previous studies (Bandelj *et al.*, 2007; Crozier *et al.*, 2007). The level of heterozygosity found in a population is highly dependent on the mating system and the evolutionary history of the species, besides a range of other factors. Although various microsatellite markers and sample sizes have been used in diversity studies on *Ficus* species, these same values made it possible for us to assume the present status of genetic variability due to the mating system and plant-insect mutualism. Pollen and diaspores in *Ficus* species are dispersed over long distances (Kinnaird *et al.*, 1996; Nason *et al.*, 1996), thereby implying that the flight distance of pollinators and dispersal range might predict high levels of genetic variation in these species (Hamrick and Loveless, 1989; Epperson and Alvarez-Buylla, 1997; Nazareno and Carvalho, 2008).

There were significant deviations from Hardy-Weinberg equilibrium (HWE) in seven *F. citrifolia* loci. As to *F. eximia*, nine loci were not in HWE (Table 1). Either the intrapopulation substructure produced by the sampling effect or the presence of null alleles may have caused these deviations, since analyses at the population level showed deviations from HWE for these same loci. Furthermore, as these results were obtained by using microsatellites developed in another species, the probability of a null allele occurring would be much higher than in the case of testing in the species from which they were isolated (Kim *et al.*, 2004). In future studies, the influence of null alleles on the transferability of microsatellite markers and their applicability in other species should be investigated.

The chi-square test for the independent segregation hypothesis indicated that all loci for *F. citrifolia* were in linkage equilibrium. As to *F. eximia*, however, significant linkage disequilibrium was found for the loci Frub38 and Frub415. The combined values for probabilities of paternity exclusion in all the 11 polymorphic loci were 0.996 for *F. citrifolia* and 0.995 for *F. eximia*. Using the 11 polymorphic loci enabled us to distinguish all the 60 *F. citrifolia* and 60 *F. eximia* individuals in two populations from southeastern Brazil. Hence, transferred microsatellite markers should allow for detailed parentage studies in natural populations, even in situations where both maternity and paternity are unknown. Moreover, these microsatellite loci could be highly useful for providing data on population genetics.

The high transferability of microsatellite markers developed from different subgenera of *Ficus* for *F. citrifolia* and *F. eximia* confirm the general applicability of *Ficus* microsatellite primers to this very large genus. Currently, we are using these markers to investigate the impact of tropical deforestation on population structure and genetic diversity in forest fragments in the Atlantic Rain Forest *sensu lato*, where *F. citrifolia* and *F. eximia* are native.

## Figures and Tables

**Table 1:** Transferability and characterization of *Ficus insipida* (prefix Fins)^1^, *Ficus racemosa* (prefix Frac)^2^ and *Ficusrubiginosa* (prefix Frub)^2^ microsatellite loci in 60 *Ficus citrifolia* and 60 *Ficuseximia* individuals from two populations in southeastern Brazil.

Locus accession n.	Primer sequence 5'-3'	Repeat motif	Size range (bp)	Characterization of microsatellite markers										
				*Ficus citrifolia*						*Ficus eximia*				
				bp_O_	*N*_a_	*T*_a_	*H*_O_*/h*_E_	Pe		bp_O_	*N*_a_	*T*_a_	*H*_O_*/h*_E_	Pe
FinsN1	F: AGGGCTGAGATAGGTTGATT	(TA)_2_(CA)_10_(TA)_7_	150-160	151-160	4	50	0.93/0.68-	0.248		158-164	4	49	0.93/0.66-	0.231
AM039805	R: TAAGTTGGTGTGTGGCATC	CATA(TG)_2_												
FinsT7	F: GAATCTGGAGGTGGAATAAAC	(TA)_11_(TG)_16_	193-210	172-182	5	46	0.17/0.31-	0.051		178	1	50	*	-
AM039810	R: AAAGATCGCTCGTCAACC													
FinsU9	F: CGTGTATTGATGTGTGTGTG	(AG)_16_	148-156	-	-	-	-	-		-	-	-	-	-
AM039811	R: TCACCTCCTCCTTCTTTTG													
Frac86	F: TGTCACTGTTCTGTTTGTGC	(TC)_13_(CA)_10_	153-167	166-196	15	46	0.90/0.91	0.662		162-178	8	50	0.25/0.83-	0.466
DQ659281	R: CAGCCAACCCTCAAGTATAAGA													
Frac110	F: CCAGAACAGGTTGGACGTAAC	(CA)_13_	166-172	-	-	-	-	-		-	-	-	-	-
DQ659282	R: GGATTACCCGCGCTATGAAGT													
Frac154	F: ACCCAAGAGCCCAAACTCGT	(AC)_13_	147-151	140	1	48	*	-		144-159	6	48	1.00/0.78-	0.380
DQ659284	R: TCAACCCTTGTGCTCCTTGC													
Frub29	F: CCACTTTGGAATGTCACTTGGA	(AG)_24_	188-226	186-228	8	48	0.73/0.78	0.399		195-255	12	50	0.72/0.87-	0.577
DQ659290	R: TGAACACGCCAACTGAGAATG													
Frub38	F: ACACGTGCAGTGCTGCTGA	(AG)_8_AAC(GA)_13_	195-255	190-220	6	50	0.67/0.77	0.359		192-222	7	49	0.36/0.78-	0.383
DQ659291	R: ACAGCTGCCCAATTCCTTGA													
Frub61	F: GTACACTCTCTTAGCTGCC	(TC)_24_	145-188	130-178	12	50	0.55/0.82-	0.468		120-165	11	50	0.64/0.87-	0.561
DQ659292	R: GTACACTCTCTTAGCTGCC													
Frub93	F: TATTTCAATAACATCTCCTCAAC	(GA)_11_	106-136	-	-	-	-	-		-	-	-	-	-
DQ659293	R: TACGTTTGTTATGGACTTTGGC													
Frub391	F: AGATGTCAAATAAGGTCAGCT	(TG)_19_	149-173	136-158	9	49	0.46/0.83-	0.485		140-156	5	50	0.95/0.77-	0.352
Q659294	R: AGATGCAGTTCCATACAATTC													
Frub415	F: GCACGTAGTCGGTGTTAAGC	(AC)_10_	150-173	132-156	4	50	0.58/0.54	0.141		158-164	2	48	0.39/0.42	0.087
DQ659297	R: CTGTGCGGAATAAAAGCTAGC													
Frub416	F: CAGCAATGATCTTGACCT	(CA)_14_ (CA)_8_	228-246	212-232	6	49	1.00/0.79-	0.391		215-230	4	49	0.93/0.64-	0.216
DQ 659289	R:ACTCATCAATATCTCTAAACAAC													
Frub422	F: GCGTGAAATTTATGCTATGA	(AC)_18_	172-190	155-176	8	49	0.48/0.80-	0.426		156-170	8	49	0.70/0.79	0.407
DQ 659298	R: GTTTCGTTCAATTTGAGTGC													
Frub436	F: GTACTGTGATTAGTATCTTTGA	(AC)_22_	135-159	133-178	9	48	0.55/0.83-	0.478		148-188	9	48	0.53/0.82-	0.462
DQ 659299	R CTAGCAATAACTCACTGATATTG													
Sum^1^/Average^2^/Cumulative probability of exclusion^3^				87^1^/7.3^2^		0.58^2^/0.67^2^	0.996^3^			77^1^/6.4^2^		0.62^2^/0.69^2^	0.995^3^	

1, Vignes *et al.* 2006; 2, Crozier *et al.* 2007; size range (bp), original data for species for which primers were developed; bp_O_*,* observed size range*; N*_a_ number of alleles; *T*_a_, annealing temperature (°C); *H*_O_, observed heterozygosity; *H*_E_, expected heterozygosity; Pe, paternity exclusion probability; *, FinsT7 and Frac154 were monomorphic in just one of the species; -, statistically significant deviation from Hardy-Weinberg equilibrium (p < 0.05).
